# ZAK Inhibitor PLX4720 Promotes Extrusion of Transformed Cells via Cell Competition

**DOI:** 10.1016/j.isci.2020.101327

**Published:** 2020-06-30

**Authors:** Takeshi Maruyama, Ayana Sasaki, Sayuri Iijima, Shiyu Ayukawa, Nobuhito Goda, Keisuke Tazuru, Norikazu Hashimoto, Takashi Hayashi, Kei Kozawa, Nanami Sato, Susumu Ishikawa, Tomoko Morita, Yasuyuki Fujita

**Affiliations:** 1 Division of Molecular Oncology, Institute for Genetic Medicine, Hokkaido University Graduate School of Chemical Sciences and Engineering, Sapporo 060-0815, Japan; 2 Waseda Institute for Advanced Study, Waseda University, Tokyo 169-8050, Japan; 3 Department of Life Science and Medical Bioscience, School of Advanced Science and Engineering, Waseda University, Tokyo 162-8480, Japan; 4 Fujii Memorial Research Institute, Otsuka Pharmaceutical Co., Ltd., Shiga 520-0106, Japan; 5 Biomedical Technology Research Center, Tokushima Research Institute, Otsuka Pharmaceutical Co., Ltd., Tokushima 771-0192, Japan; 6 Department of Molecular Oncology, Graduate School of Medicine, Kyoto University, Kyoto, 606-8501, Japan

**Keywords:** Chemistry, Biological Sciences, Cancer

## Abstract

Previous studies have revealed that, at the initial step of carcinogenesis, transformed cells are often eliminated from epithelia via cell competition with the surrounding normal cells. In this study, we performed cell competition-based high-throughput screening for chemical compounds using cultured epithelial cells and confocal microscopy. PLX4720 was identified as a hit compound that promoted apical extrusion of RasV12-transformed cells surrounded by normal epithelial cells. Knockdown/knockout of ZAK, a target of PLX4720, substantially enhanced the apical elimination of RasV12 cells *in vitro* and *in vivo*. ZAK negatively modulated the accumulation or activation of multiple cell competition regulators. Moreover, PLX4720 treatment promoted apical elimination of RasV12-transformed cells *in vivo* and suppressed the formation of potentially precancerous tumors. This is the first report demonstrating that a cell competition-promoting chemical drug facilitates apical elimination of transformed cells *in vivo*, providing a new dimension in cancer preventive medicine.

## Highlights

• A cell competition-based high-throughput screen identifies chemical enhancers
• PLX4720 enhances the apical elimination of RasV12 cells via ZAK inhibition
• ZAK negatively modulates cell competition regulators
• PLX4720 treatment promotes apical elimination of RasV12 cells *in vivo*

## Graphical Abstract

**Figure:**
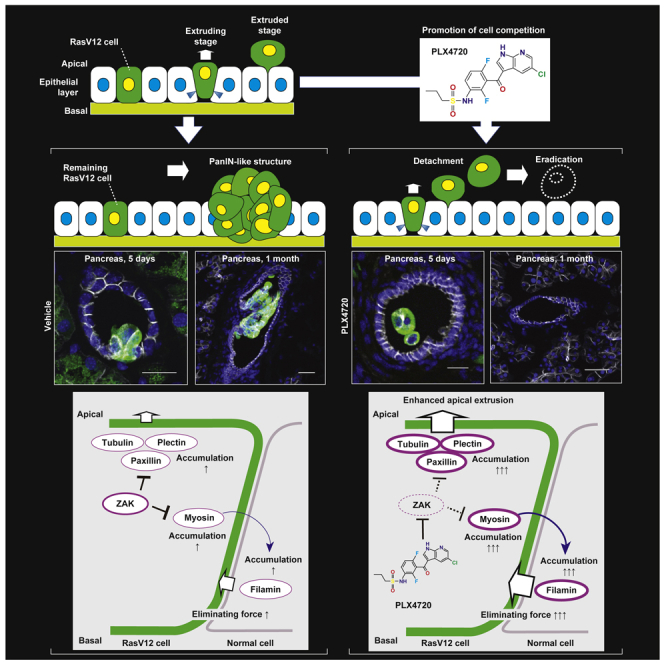

## Introduction

Despite extensive efforts in chemotherapeutic research, cancers are often resistant to chemical drugs. In addition, certain types of malignant tumors such as lung and pancreatic cancers are incurable even in the early stage detection (Jemal et al., 2006; Siegel et al., 2018). Recent studies using next-generation sequencing technology have revealed that, in our body, there are a number of abnormal lesions; although they apparently look normal, they comprise focally accumulated transformed cells that harbor just one or two oncogenic mutations (Martincorena et al., 2015, 2018). Hence, to overcome cancer, an alternative strategy would be a preventive cure: prophylactically eradicating such potentially pre-cancerous lesions.

Our recent studies have demonstrated that, at the initial stage of carcinogenesis, normal and transformed epithelial cells often compete with each other for survival: a phenomenon called cell competition (Maruyama and Fujita, 2017). For instance, when oncoprotein Ras- or Src-transformed cells are surrounded by normal epithelial cells, the transformed cells are extruded from the apical surface of the normal epithelial monolayer (Hogan et al., 2009; Kajita et al., 2010). These apically extruded transformed cells will eventually be eliminated from epithelial tissues and expelled outside the body (Kon et al., 2017; Sasaki et al., 2018), implying that this is a cancer preventive mechanism. During this cell competition process, normal epithelial cells first recognize the presence of neighboring transformed cells (Hogan et al., 2009). Normal cells then accumulate the cytoskeletal protein Filamin at the boundary with transformed cells, thereby generating physical forces and actively eliminating transformed cells from epithelia: a mechanism called epithelial defense against cancer (EDAC) (Kajita et al., 2014). On the other hand, the surrounded transformed cells also react to normal cells and accumulate a set of cytoskeletal components (Kadeer et al., 2017; Kasai et al., 2018; Ohoka et al., 2015; Saitoh et al., 2017). For example, Paxillin, Plectin, and Tubulin form a complex and accumulate at the apical side of transformed cells (Kadeer et al., 2017; Kasai et al., 2018). These three proteins mutually influence their non-cell-autonomous accumulation, which also plays a crucial role in apical extrusion of RasV12-transformed cells.

To identify chemical compounds that promote cell competition between normal and transformed cells, we have established a high-throughput screening platform based on epifluorescent microscopy and identified Rebeccamycin as one of the hit compounds (Yamauchi et al., 2015). Rebeccamycin and its derivatives promote apical extrusion of transformed cells *in vitro*. However, these chemical compounds could not be applied to *in vivo* analyses because of their high cytotoxicity. In this study, we have newly established a confocal microscopy-based screening system and identified several chemical compounds that promote apical elimination of RasV12-transformed cells from epithelia *in vitro* and *in vivo*. This study will elicit future interest to establish cancer-preventive therapy by targeting cell competition.

## Results

### PLX4720 and Its Derivatives Promote Apical Elimination of RasV12-Transformed Cells

**Figure 1:**
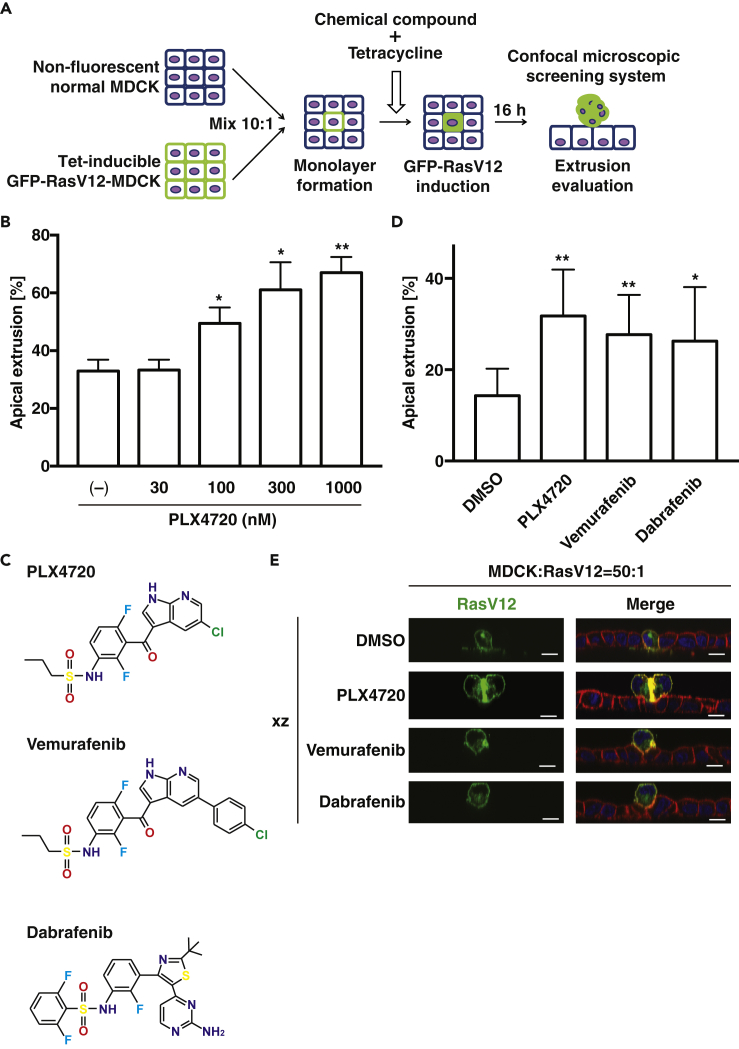
Cell Competition-Based High-Throughput Screening for Chemical Compounds Using Confocal Microscopy (A) A scheme of cell competition-based screening. (B) The dose-dependent effect of PLX4720 on apical extrusion of RasV12-transformed cells. (C) Chemical structure of PLX4720 and its derivative compounds. (D and E) The effect of PLX4720 and its derivative compounds (1 μM) on apical extrusion of RasV12-transformed cells. (B, D, and E) MDCK-pTR GFP-RasV12 cells were mixed with normal MDCK cells on collagen gels. Cells were cultured with the indicated chemical compounds and fixed after 16 h incubation with tetracycline and stained with phalloidin (red) and Hoechst (blue). (B and D) Quantification of apical extrusion of RasV12 cells. n ≧ 100 cells for each experimental condition. Data are mean ± SD from three independent experiments. ∗p < 0.05, ∗∗p < 0.01 (Student's t tests). (E) Representative XZ images of normal and RasV12 cells. Scale bars: 10 μm.

To identify chemical compounds that promote apical extrusion of RasV12-transformed cells, we have optimized the previously established screening system (Yamauchi et al., 2015) by employing a confocal microscopy-based high-throughput platform (Figure 1A). First, normal MDCK cells and MDCK-pTR GFP-RasV12 cells were mixed at a ratio of 10:1 and cultured in a collagen-coated 96-well plate until they formed an epithelial monolayer. Then, the cells were incubated for 16 h with a small chemical compound, together with tetracycline to induce expression of GFP-RasV12. In this study, we used a chemical compound library consisting of various kinase inhibitors. Finally, apically extruded GFP-RasV12 cells were captured by confocal microscopic analyses (Figure S1A). After the primary screening, we obtained PLX4720 as a hit compound (Figure S1B). PLX4720 substantially enhanced apical extrusion of RasV12 cells that were surrounded by normal cells (Figure S1B), but not increased apical elimination of RasV12 cells that were cultured alone (Figure S1C). In addition, PLX4720 promoted apical extrusion in a dose-dependent manner (Figure 1B). Previous reports have demonstrated that PLX4720 strongly inhibits the activity of both ZAK and the active mutant of Raf (BRAF-V600E) but shows a much weaker inhibitory effect on wild-type Raf (Table S1) (Fabian et al., 2005; Karaman et al., 2008; Vin et al., 2013). ZAK, also called MLTK (MLK-like mitogen-activated protein triple kinase), is a member of the serine-threonine kinase MAPKKK family and is involved in osmotic stress response (Gotoh et al., 2001), but its role in cell competition has not been studied yet. We then found that other structurally related, ZAK-inhibiting compounds Vemurafenib and Dabrafenib significantly enhanced apical elimination of MDCK-pTR GFP-RasV12 cells from a monolayer of normal MDCK cells (Figures 1C–1E and Table S1). MDCK cells do not harbor V600E mutations in the *BRAF* gene locus. In addition, an inhibitor of the BRAF downstream kinase MEK suppresses apical elimination of RasV12-transformed cells (Hogan et al., 2009). Thus, it is plausible that the effect of these compounds on apical extrusion of RasV12 cells is attributed to inhibition of ZAK, rather than that of Raf.

### ZAK Is a Negative Regulator for Apical Extrusion of RasV12-Transformed Cells

**Figure 2:**
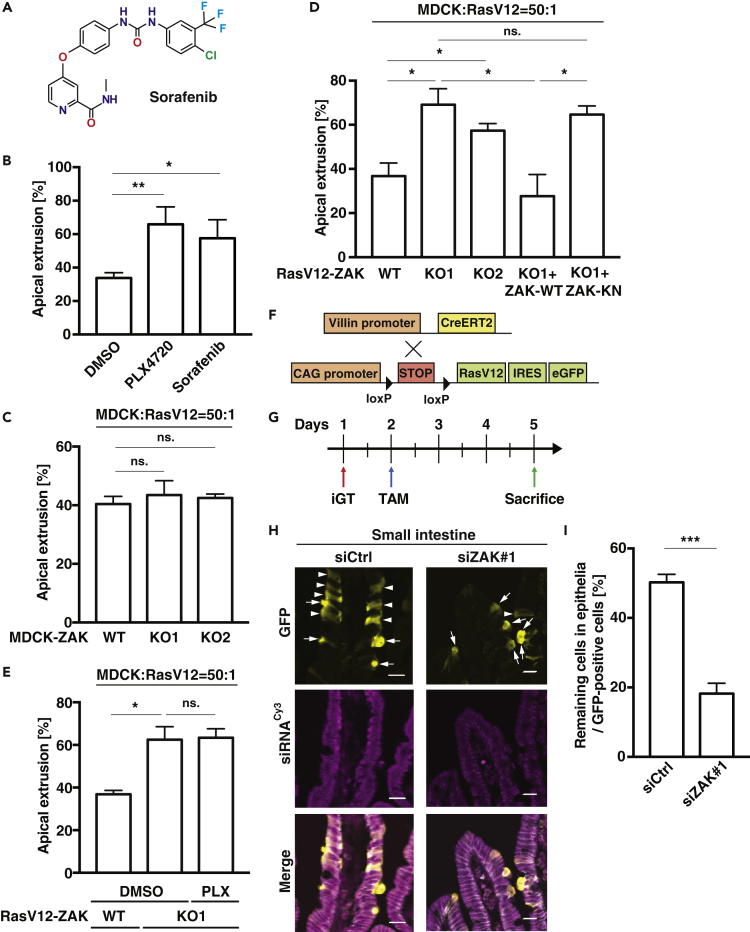
ZAK Negatively Regulates Apical Extrusion of RasV12-Transformed Cells *In Vitro* and *In Vivo* (A) The chemical structure of Sorafenib. (B) Sorafenib, another ZAK inhibitor, promotes apical extrusion of RasV12-transformed cells. MDCK-pTR GFP-RasV12 cells were mixed with normal MDCK cells on collagen gels. The cells were fixed after 24 h incubation with tetracycline together with DMSO, PLX4720, or Sorafenib (1 μM). (C) Knockout of ZAK in normal cells does not affect the efficiency of apical extrusion. MDCK-pTR GFP-RasV12 cells were mixed with normal MDCK ZAK-WT or -KO cells on collagen gels. The cells were fixed after 24 h incubation with tetracycline, and apical extrusion was quantified. (D) Knockout of ZAK in RasV12-transformed cells promotes apical extrusion. MDCK-pTR GFP-RasV12 ZAK-wild-type (WT), ZAK-knockout (KO), ZAK-KO + HA-ZAK-WT, or ZAK-KO + HA-ZAK-kinase-negative (KN) cells were mixed with normal MDCK cells on collagen gels. The cells were fixed after 24 h incubation with tetracycline, and apical extrusion was quantified. (B–D) n ≧ 100 cells for each experimental condition. Data are mean ± SD from three independent experiments. ∗p < 0.05, ∗∗p < 0.01, ns.: not significant (Student's t tests). (E) PLX4720 does not enhance the extrusion efficiency of ZAK-knockout RasV12 cells. MDCK-pTR GFP-RasV12 ZAK-wild-type (WT) or ZAK-KO cells were mixed with normal MDCK cells on collagen gels. The cells were fixed after 24 h incubation with PLX4720 and tetracycline, and the frequency of apical extrusion was quantified. n≧100 cells for each experimental condition. Data are mean ± SD from three independent experiments. ∗p < 0.05, ns.: not significant (Student's t tests). (F)Strategy for the establishment of the cell competition mouse model using an intestine-specific Villin-CreERT2 system. (G) Experimental design for short-term tamoxifen (TAM) administration on the small intestine after siRNA introduction by electroporation-based intestinal gene transfer (iGT). (H and I) Knockdown of ZAK promotes apical extrusion within intestinal epithelia. (H) Immunofluorescence images of RasV12-transformed cells in the epithelium of the small intestine. The intestine tissue samples with Control-siRNA (siCtrl) or ZAK-siRNA#1 (siZAK#1) were stained with anti-GFP (yellow) and anti-E-cadherin (gray) antibodies and Cy3 (magenta). Scale bars: 20 μm. (I) Quantification of apical extrusion of RasV12 cells in the small intestine. siCtrl 345 transformed cells from three mice; siZAK#1 280 transformed cells from three mice. ∗∗∗p < 0.005 (chi-square test).

These three compounds share a similar chemical structure (Figure 1C) that is, at least partly, involved in the occupancy of the ATP pocket of the ZAK kinase domain (Mathea et al., 2016). Therefore, we tested a structurally distinct ZAK inhibitor Sorafenib (Figure 2A) and found that addition of Sorafenib also substantially promoted apical extrusion of RasV12 cells (Figure 2B) (Vin et al., 2014). These results suggest that ZAK plays a negative role in the elimination of transformed cells. To validate a functional role of ZAK, we depleted ZAK either in normal or RasV12-transformed cells using CRISPR-Cas9 technology and successfully generated homozygous ZAK-knockout cells, which possess 2 base-depletion (ZAK-KO1) or 17 base-insertion (ZAK-KO2). ZAK knockout in normal cells did not affect the frequency of extrusion (Figures 2C and S2A). In contrast, ZAK knockout in RasV12-transformed cells significantly enhanced apical extrusion (Figures 2D and S2B). Exogenous expression of wild-type (WT) ZAK rescued the phenotype but that of kinase-negative ZAK did not (Figures 2Dl, S2B, and S2C), suggesting a crucial role of ZAK kinase activity. Accordingly, apical extrusion of ZAK-knockout RasV12 cells was not affected by PLX4720 (Figures 2E and S2D). These results indicate that the kinase activity of ZAK in RasV12 cells negatively regulates apical extrusion. To further investigate the prevalent role of ZAK in elimination of transformed cells, we examine the effect of ZAK knockdown *in vivo* using the mouse cell competition model system (Villin-CreERT2; LSL-RasV12-IRES-eGFP) (Figure 2F) (Kon et al., 2017). To induce ZAK knockdown *in vivo*, we used the iGT (intestine-specific gene transfer) system by which short interfering RNA (siRNA) can be introduced into mouse intestinal epithelia using electroporation (Imajo et al., 2015). First, we conducted *in vivo* electroporation with control- or ZAK-siRNA, and then a low dose of tamoxifen was administered to induce the expression of the RasV12 protein in a mosaic manner within intestinal epithelia (Figure 2G) (Kon et al., 2017). The introduction of ZAK-siRNA#1 diminished the expression of ZAK (Figures S2E and S2F) and significantly promoted apical elimination of RasV12-expressing cells from the epithelium (Figures 2H and 2I). Collectively, these results demonstrate that ZAK is a crucial negative regulator for apical extrusion of RasV12-transformed cells from epithelia *in vitro* and *in vivo*.

### ZAK Negatively Modulates Cell Competition Regulators

**Figure 3:**
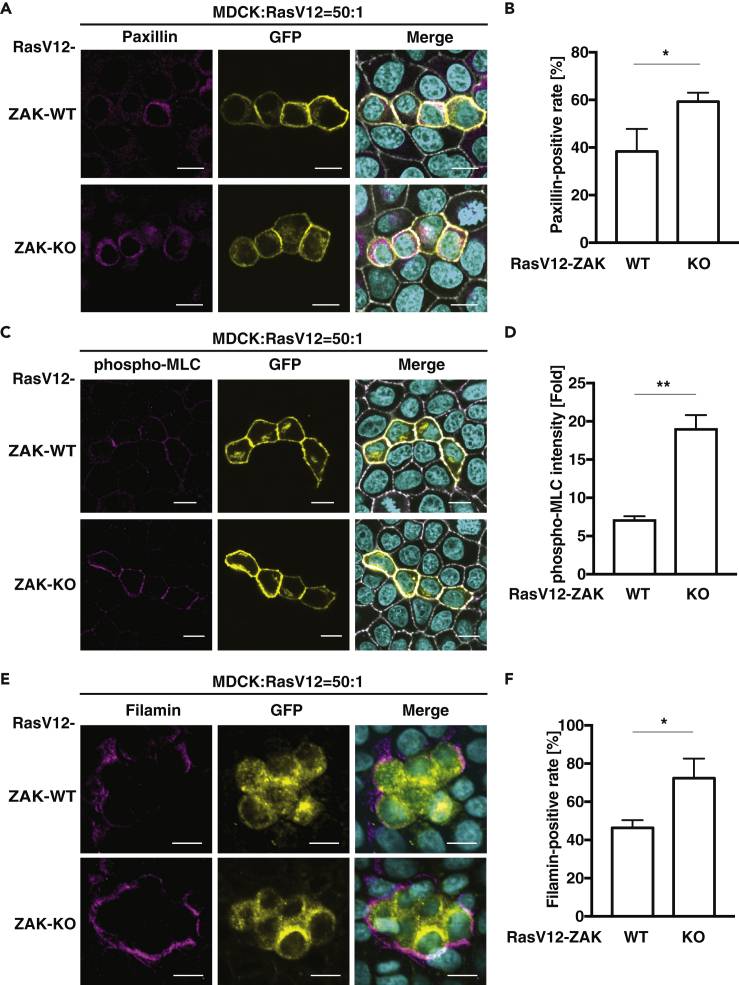
ZAK Negatively Modulates Cell Competition Regulators (A–D) ZAK-KO promotes accumulation of Paxillin (A and B) or phosphorylation of light chain of Myosin-II (phospho-MLC) in RasV12 cells surrounded by normal cells. MDCK-pTR GFP-RasV12 ZAK-wild-type (WT) or ZAK-knockout (KO) cells were mixed with normal MDCK cells on collagen gels. The cells were fixed after 16 h incubation with tetracycline and stained with anti-Paxillin (A and B) or phospho-MLC (C and D) antibody (magenta), Alexa-Fluor-647-phalloidin (gray), and Hoechst (cyan). The positive rate of Paxillin staining was quantified. For phospho-MLC quantification, the fluorescence intensity at the boundary between respective RasV12 cells and normal cells were expressed as fold change relative to the average fluorescence intensity between the surrounding normal cells. n ≧ 100 cells for each experimental condition. Data are mean ± SD from three independent experiments. ∗p < 0.05, ∗∗p < 0.01 (Student's t tests). (E and F) ZAK-KO in RasV12 cells promotes accumulation of Filamin in the neighboring normal cells. MDCK-pTR GFP-RasV12 wild-type (WT) or ZAK-knockout (KO) cells were mixed with normal MDCK cells on collagen gels. The cells were fixed after 16 h incubation with tetracycline and stained with anti-Filamin antibody (magenta) and Hoechst (cyan) (E). The positive rate of Filamin accumulation in surrounding normal cells was quantified (F). n ≧ 100 cells for each experimental condition. Data are mean ± SD from three independent experiments. ∗p < 0.05 (Student's t tests).

At the interface between normal and RasV12-transformed cells, various non-cell autonomous changes occur in both normal and transformed cells. For instance, in RasV12-transformed cells, the adaptor protein Paxillin is accumulated, and the activity of Myosin-II is elevated (Hogan et al., 2009; Kasai et al., 2018). In addition, in normal cells, the cytoskeletal protein Filamin is accumulated at the boundary with transformed cells (Kajita et al., 2014). Importantly, accumulation or activation of these molecules, in concert, positively regulate apical extrusion of transformed cells. We thus investigated the effect of ZAK knockout in Ras-transformed cells on these processes. We observed that ZAK knockout significantly promoted the accumulation of Paxillin (Figures 3A and 3B). In addition, the level of phospho-Myosin light chain (phospho-MLC), which reflects the activity of Myosin-II, increased in the ZAK-knockout transformed cells (Figures 3C and 3D). Moreover, ZAK knockout promoted the accumulation of Filamin in normal cells at the interface with Ras-transformed cells (Figures 3E and 3F). Consistently, addition of PLX4720 also facilitated the phosphorylation of MLC and the accumulation of Filamin (Figure S3). These findings suggest that ZAK negatively modulates the cell competition regulators, thereby suppressing apical extrusion of transformed cells.

### PLX4720 Treatment Promotes Apical Elimination of RasV12-Transformed Cells *In Vivo*

**Figure 4:**
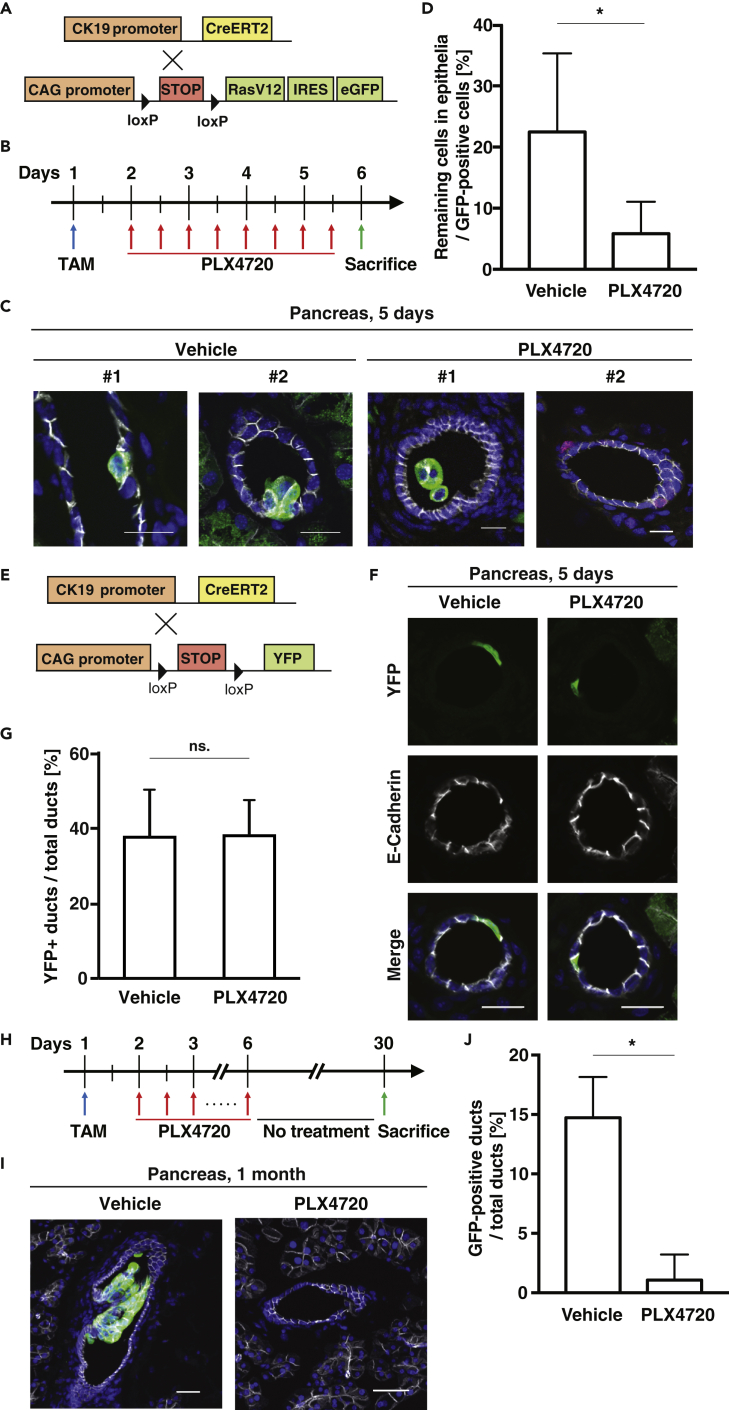
PLX4720 Treatment Suppresses Apical Extrusion of RasV12-Transformed Cells from Pancreatic Ductal Epithelia (A and E) The cell competition mouse model using a CK19-CreERT2 system and control YFP mouse. (B and H) Experimental designs for short-term (B) or long-term (H) Tamoxifen (TAM)-PLX4720 administration. (C and I) Immunofluorescence images of RasV12-transformed cells in the epithelium of the pancreatic ducts upon short-term (C) or long-term (I) vehicle or PLX4720 treatment. The tissue samples were stained with anti-GFP (green) and anti-E-cadherin (gray) antibodies and Hoechst (blue). Scale bars: 20 μm. (D) Quantification of apical extrusion of RasV12 cells for (C). Vehicle 86 cells from four mice; PLX4720 153 cells from five mice. ∗p < 0.05 (chi-square test). (F) Immunofluorescence images of YFP-expressing cells in the epithelium of the pancreatic ducts with or without PLX4720 treatment after low-dose TAM administration. The tissue samples were stained with anti-GFP (green) and anti-E-cadherin (gray) antibodies and Hoechst (blue). Scale bars: 20 μm. (G) Quantification of remaining YFP-expressing cells within epithelia for (F). Vehicle 196 cells from four mice; PLX4720 204 cells from four mice. Data are mean ± SD from four independent mice. ns.: not significant (chi-square test). (I and J) (J) Quantification of apical extrusion of RasV12 cells for (I). Vehicle 584 ducts from five mice; PLX4720 500 ducts from four mice. ∗p < 0.05 (chi-square test).

Finally, using the cell competition mouse model, we examined the effect of PLX4720 on elimination of transformed cells *in vivo*. The oncogenic mutation in the *Ras* gene occurs at the initial stage of pancreatic cancer and is involved in the formation of pancreatic intraepithelial neoplasia (PanIN), precancerous lesions in the pancreas (Bardeesy and DePinho, 2002; Morris et al., 2010). Thus, we evaluated the extrusion efficiency within the epithelia of pancreatic ducts. To monitor the fate of newly emerging RasV12-transformed cells in ductal epithelia of the pancreas, we crossed LSL-RasV12-IRES-EGFP mice with cytokeratin 19 (CK19) (epithelial-specific marker)-Cre-ERT2 mice (Figure 4A). According to the remaining level of PLX4720 in the pancreas after oral administration (Figure S4), 300 mg/kg PLX4720 was administered twice per day (Figure 4B). As previously reported (Sasaki et al., 2018), when PLX4720 was not administered, GFP-positive RasV12-expressing cells often remained within epithelia (Figures 4C and 4D). In contrast, after 5 days of PLX4720 administration, most of RasV12-expressing cells were apically detached into the ductal lumen or absent within the pancreatic ducts (Figures 4C and 4D). As a control, YFP-expressing cells just remained in the epithelia, and PLX4720 did not affect the rate of YFP-positive cells in the pancreatic ducts (Figures 4E–4G). After 1 month of tamoxifen treatment, some remaining RasV12 cells proliferated and formed a PanIN-like tumorous structure (Figures 4H and 4I). But, PLX4720 administration profoundly decreased the number of remaining RasV12-expressing cells (Figures 4I and 4J). Collectively, these results demonstrate that PLX4720 treatment promotes apical elimination of RasV12-transformed cells *in vivo* and suppresses the formation of potentially precancerous tumors.

## Discussion

Pancreatic cancer is one of the major causes of cancer-associated mortality, and the prognosis has remained seriously poor. The most common mutations are *KRAS, TP53*, and *SMAD4*, none of which are, however, currently druggable targets (Kleeff et al., 2016). Therefore, a novel approach for early diagnosis and preventive treatment at the curable stage would be desired. In this study, we have identified chemical compounds that enhance apical elimination of RasV12-transformed cells, a cancer preventive process. Especially, PLX4720 suppresses the formation of pre-cancerous tumors within the pancreatic ducts. The number of the apically delaminated RasV12 cells decreases as time goes by after PLX4720 administration, suggesting that the apically detached cells would undergo anoikis or be eradicated outside the body. Thus, PLX4720 treatment would contribute to a decreased risk of carcinogenesis. This is the first report demonstrating that a cell competition-promoting chemical drug facilitates apical elimination of transformed cells *in vivo*.

Previous studies have demonstrated that PLX4720 strongly inhibits the activity of both ZAK and the active mutant of Raf (BRAF-V600E) but not that of wild-type Raf (Fabian et al., 2005; Karaman et al., 2008; Vin et al., 2013). Instead, PLX4720 can cause paradoxical activation of wild-type Raf (Hatzivassiliou et al., 2010; Poulikakos et al., 2010). Indeed, the slight activation of ERK is observed upon PLX4720 treatment in MDCK cells (data not shown), implying that PLX4720 induces attenuation of ZAK activity as well as ERK activation, which might promote apical elimination of transformed cells in an orchestrated fashion. Additionally, the involvement of other kinases, the activity of which could be potentially inhibited by ZAK inhibitors, cannot be ruled out (Karaman et al., 2008; Vin et al., 2013). However, a structurally distinct ZAK inhibitor Sorafenib or ZAK knockout enhances apical extrusion, indicating that suppression of ZAK alone is sufficient to regulate this process. ZAK is a family member of MAPKKK (Gotoh et al., 2001), but its function remains enigmatic. We demonstrate that ZAK is a novel key regulator for cell competition between normal and RasV12-transformed cells. ZAK activity in RasV12 cells plays a negative role in their apical extrusion by modulating the activity or localization of downstream cell competition regulators: Paxillin and Myosin-II in RasV12 cells and filamin in normal cells. However, it remains to be uncovered what are the direct substrate proteins of ZAK in this process. ZAK is reported to upregulate the activity of ERK, JNK, and p38 upon various stimuli such as inflammation and osmotic stress (Gotoh et al., 2001; Wong et al., 2013; Yang et al., 2010). Further studies would be required to understand more detailed molecular mechanisms of this ZAK-regulated cell extrusion.

A previous study using a cell competition mouse model has revealed that obesity and obesity-induced chronic inflammation diminish apical elimination of RasV12-transformed cells in the small intestine and pancreas (Sasaki et al., 2018). These environmental conditions affect apical elimination of transformed cells, leading to the formation of precancerous lesions. In addition, inflammation can induce ZAK-mediated activation of JNK and p38 (Wong et al., 2013). Given that the inflammation suppresses apical extrusion (Sasaki et al., 2018) and activates the ZAK-JNK/p38 pathway, it is plausible that obesity-induced inflammation suppresses elimination of transformed cells, at least partly, through modulation of the ZAK-JNK/p38 pathway. It will be tested whether the PLX4720 treatment can promote the extrusion of the remaining RasV12-transformed cells under these environmental conditions.

A recent study demonstrates that cell competition can occur during the process of carcinogenesis in human (Madan et al., 2019). Moreover, several lines of studies have revealed that there are a number of focally colonized transformed cells in our bodies and that these precancerous lesions progressively accumulate with age. Hence, this study would shed light on those clinically unexplored lesions and provide a new dimension in cancer preventive medicine. Further elucidation of cell competition mechanisms would pave a way to cancer prophylactic treatment.

### Limitations of the Study

Our study has some limitations. First, it remains to be uncovered what the direct substrate proteins of ZAK are. Second, to promote accumulation of Myosin-II, the substrate of ZAK should regulate Myosin-II accumulation. In this case, the substrate needs to be identified. In addition, it is also a possibility that direct regulation of Myosin-II by ZAK contributes to promoting stiffness of RasV12 cells by accumulation of Myosin-II at the boundary. Last, it is required that PanIN-like structure is related to tumorigenesis and the PLX is further analyzed in-depth using tumor growth in the xenograft model.

### Resource Availability

#### Lead Contact

Further information and requests for resources and reagents should be directed to and will be fulfilled by the Lead Contact, Takeshi Maruyama (tmaru@aoni.waseda.jp).

#### Materials Availability

Materials generated in this study are available from the corresponding author on requests.

#### Data and Code Availability

The original dataset generated during this study will be publicly available.

Data: https://doi.org/10.17632/m296vz4d9d.1.

## Methods

All methods can be found in the accompanying Transparent Methods supplemental file.

## Supplemental Information

Document S1. Transparent Methods, Figures S1–S4, and Tables S1 and S2
