# Supplementary material for: ZAK Inhibitor PLX4720 Promotes Extrusion of Transformed Cells via Cell Competition
Source: iScience. 2020 Jun 30;23(7):101327. doi: 10.1016/j.isci.2020.101327 (PMC7371749; doi:10.1016/j.isci.2020.101327)
Supplement: Document S1. Transparent Methods, Figures S1–S4, and Tables S1 and S2 [file mmc1.pdf]

iScience, Volume *23*

Supplemental Information

ZAK Inhibitor PLX4720 Promotes Extrusion of Transformed Cells via Cell Competition

Takeshi Maruyama, Ayana Sasaki, Sayuri Iijima, Shiyu Ayukawa, Nobuhito Goda, Keisuke Tazuru, Norikazu Hashimoto, Takashi Hayashi, Kei Kozawa, Nanami Sato, Susumu Ishikawa, Tomoko Morita, and Yasuyuki Fujita

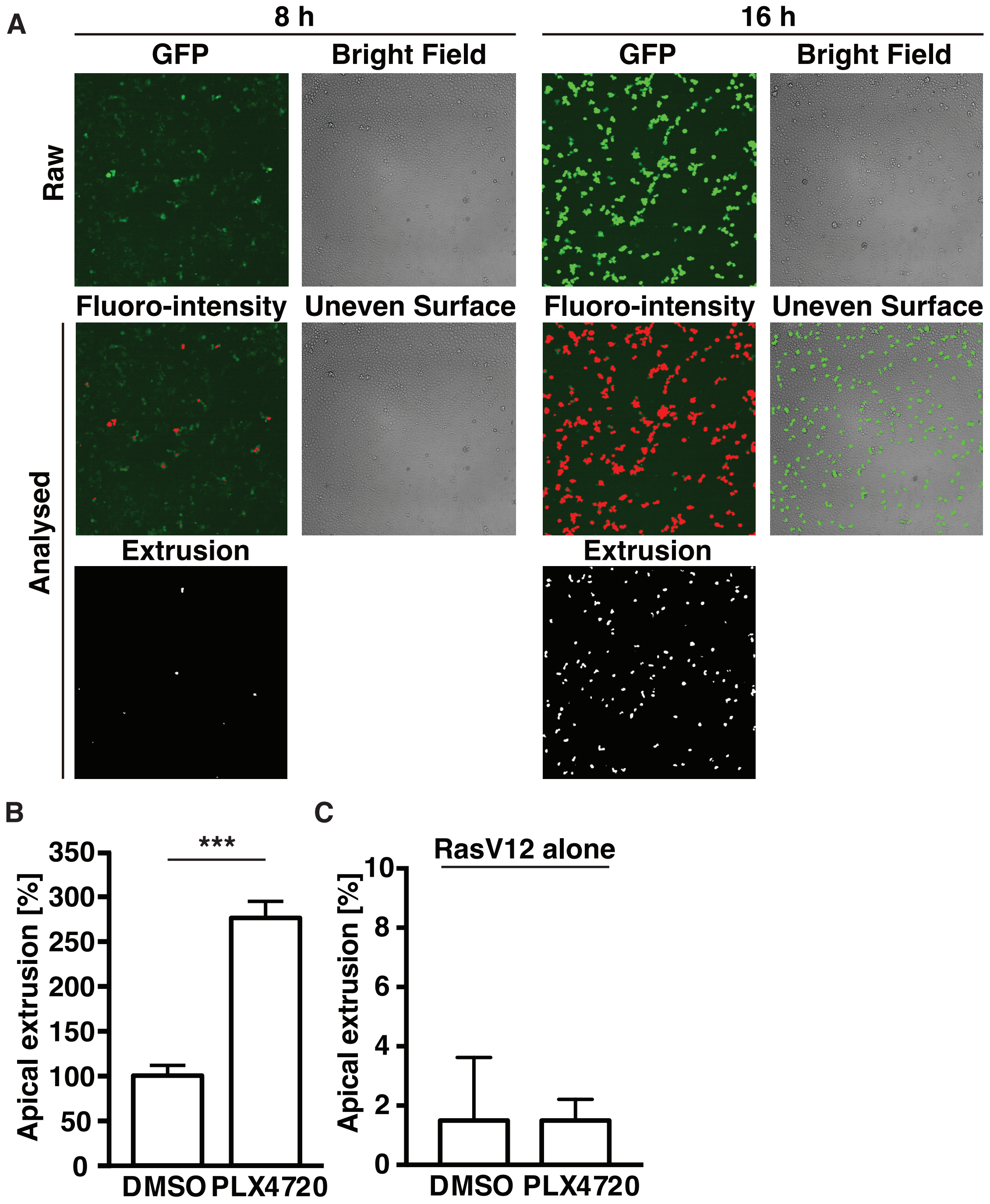

Figure S1. The establishment of a high-throughput screening platform using confocal microscopy, related to Figure 1 (A) MDCK cells were mixed with MDCK-pTR GFP-RasV12 cells and seeded into a collagen-coated 96-well plate. The mixed cells were incubated until a monolayer was formed. Then, the culture medium was exchanged for new medium containing 10 μg/ml tetracycline, followed by incubation for 16 h. Cells were fixed and stained with Hoechst 33342 and Alexa555-Phalloidin. The images of cells were captured using a confocal imaging analyzer, and GFP-fluorescent and Bright field images were acquired. The GFP-positive cells were selected by GFP intensities as shown in red in the panel 'Analysed-Fluoro-intensity'. The uneven surface area was independently analysed using a 65536-gradient step bright-field and shown as light green in the panel 'Analysed-Uneven Suface'. Double positive cells are extracted as white spots as shown in the panel 'Analysed-Extrusion' and defined as apically extruded RasV12 cells. (B) The validation assay showing the effect of 10 μM PLX4720 on apical extrusion of RasV12 cells. Data are mean ± SD from three independent experiments. ***P<0.005 (Student's *t*-tests). (C) PLX4720 did not affect the RasV12-transformed cells in the condition of RasV12 alone. Data are mean ± SD from three independent experiments.

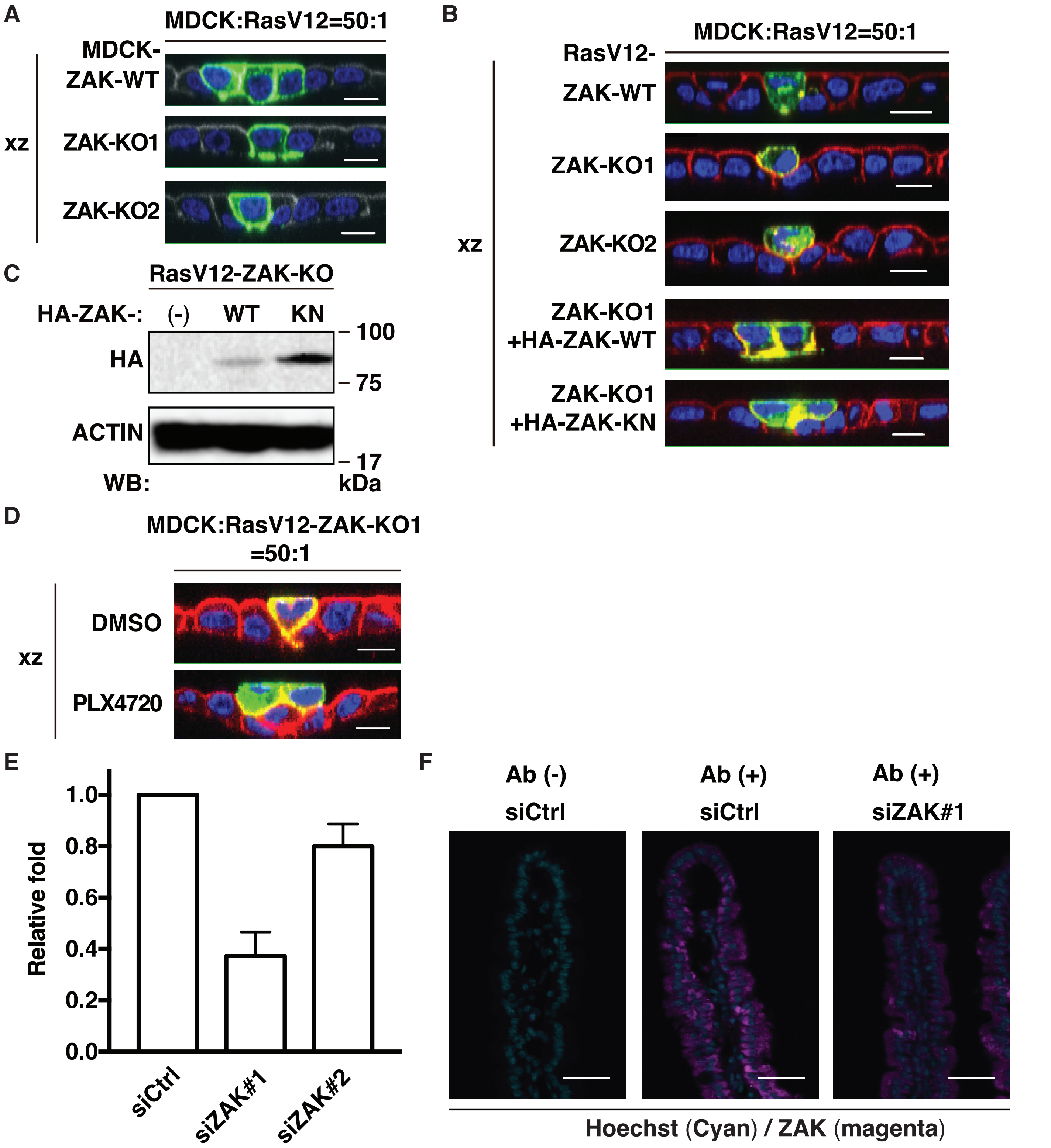

Figure S2. Supplementary figures for Figure 2

ZAK-knockout in RasV12-transformed cells promotes apical extrusion (A) Representative XZ images for Figure 2c. Phalloidin and Hoechst staining are shown in grey and blue, respectively. Scale bars: 10 μm. (B) MDCK-pTR GFP-RasV12 ZAK-wild-type (WT) cells or ZAK-KO cells constitutively expressing HA-ZAKα-WT or -KN were mixed with normal MDCK cells on collagen gels. The cells were fixed after 24 h incubation with tetracycline and stained with Alexa-Fluor-568-phalloidin (red) and Hoechst (blue). (C) Establishment of MDCK-pTR GFP-RasV12 ZAK-KO cells stably expressing wild-type (WT) or kinase-negative (KN) ZAK. Expression of exogenous ZAK proteins was examined by western blotting using anti-HA antibody. (D) Representative XZ images for the Figure 2d. (C and D) Scale bars: 10 μm.

Knockdown of ZAK in the mouse intestinal epithelium using iGT (E) ZAK-knockdown efficiency of mouse embryonic fibroblasts (MEFs). MEFs were transfected with Control-siRNA (siCtrl), ZAK-siRNA#1 (siZAK#1) or ZAK-siRNA#2 (siZAK#2). After 48 h, the transfected MEFs were subjected to qPCR. Data are mean ± SD from two independent experiments. (F) Immunofluorescence images of ZAK in the epithelium of the small intestine after iGT. The tissue samples were stained with anti-ZAK (magenta) antibody and Hoechst (cyan). Scale bars: 20 μm.

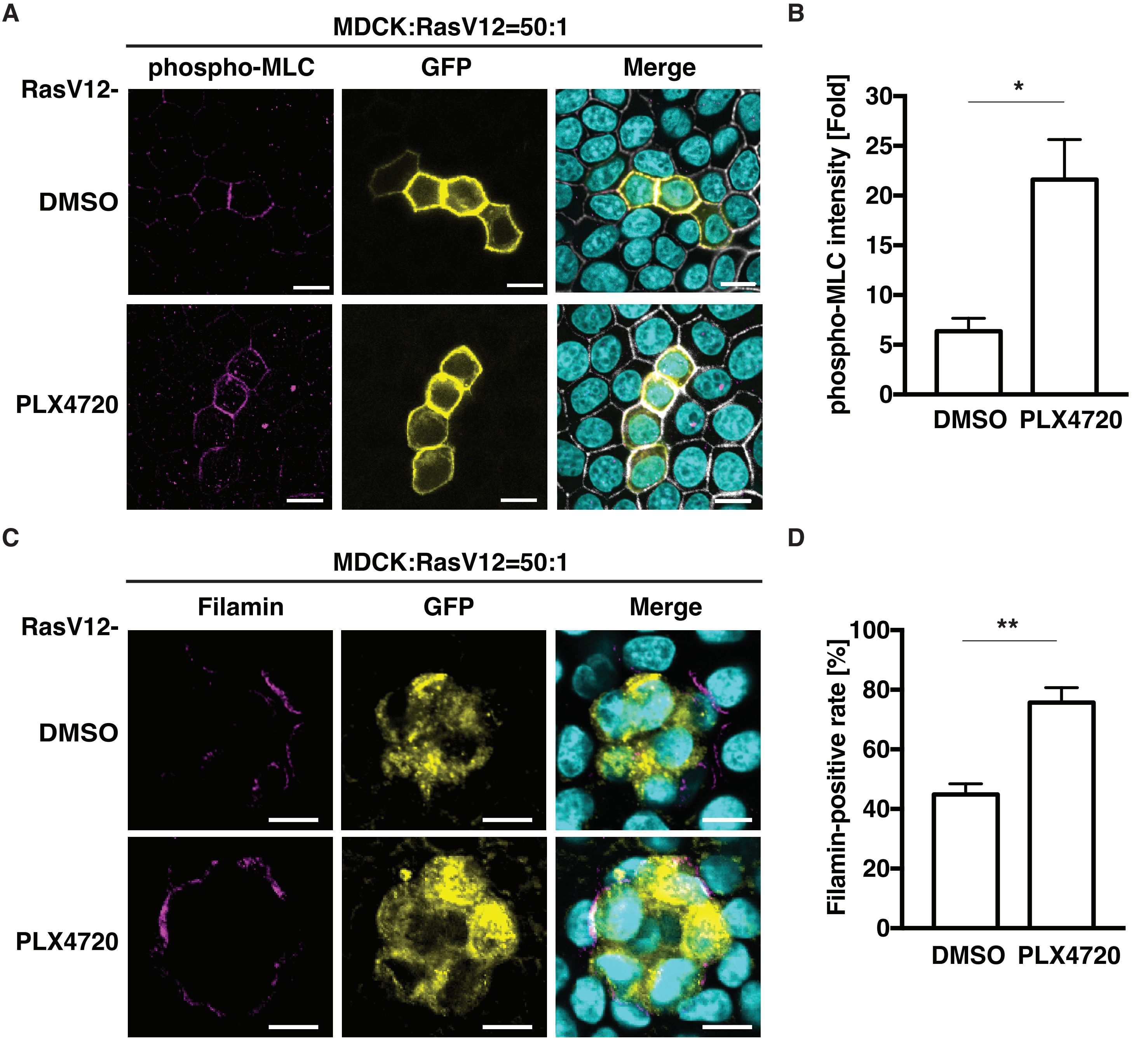

Figure S3. Supplementary figures for Figure 3

(A and B) PLX4720 promotes phosphorylation of light chain of Myosin-II (phospho-MLC) in RasV12 cells surrounded by normal cells. MDCK-pTR GFP-RasV12 cells were mixed with normal MDCK cells on collagen gels. The cells were fixed after 16 h incubation with PLX4720 (1 μM) and tetracycline, and stained with anti-phospho-MLC antibody (magenta), Alexa-Fluor-647-phalloidin (gray) and Hoechst (cyan). For phospho-MLC quantification, the fluorescence intensity at the boundary between respective RasV12 cells and normal cells were expressed as fold change relative to the average fluorescence intesity between the surrounding normal cells. n ≧ 100 cells for each experimental condition. Data are mean ± SD from three independent experiments. $^*P < 0.05$ (Student's t-tests). (C and D) PLX treatment promotes accumulation of Filamin in the neighboring normal cells. MDCK-pTR GFP-RasV12 cells were mixed with normal MDCK cells on collagen gels. The cells were fixed by methanol after 16 h incubation with PLX4720 (1 μM) and tetracycline, and stained with anti-Filamin antibody (magenta) and Hoechst (cyan). The positive rate of Filamin accumulation in surrounding normal cells was quantified. n ≧ 100 cells for each experimental condition. Data are mean ± SD from three independent experiments. $^{**}P < 0.01$ (Student's t-tests).

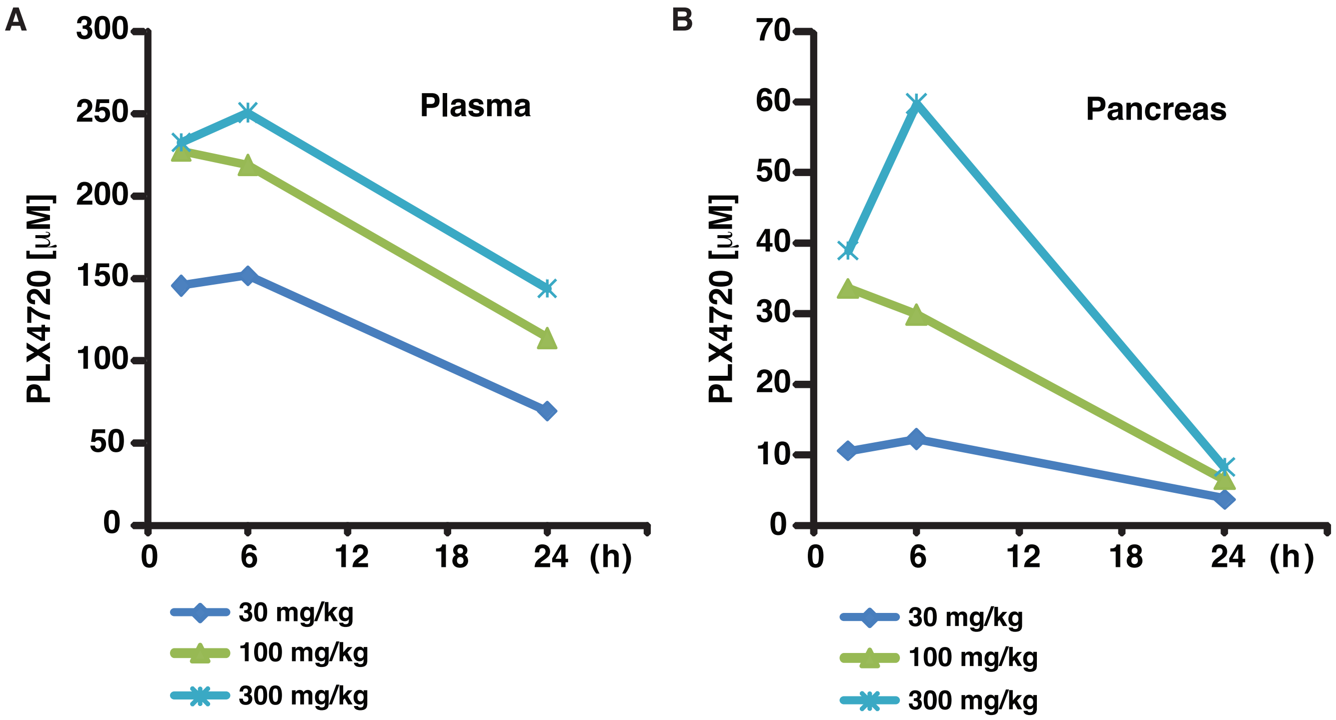

Figure S4. Confirmation of PLX4720-remaining levels, related to Figure 4 (A and B) The remaining PLX4720 in Plasma and Pancreas. The indicated amount of PLX4720 was administered. At the indicated time points after administration, the blood and pancreas were collected. Then, the remaining amounts of PLX4720 in these collected samples were analyzed by mass spectrometry.

	ZAK		BRAFwt		BRAF V600E		ABL1	
	Observed (nM)	Range (nM)	Observed (nM)	Range (nM)	Observed (nM)	Range (nM)	Observed (nM)	Range (nM)
**PLX4720**	9.47[#18], 41[#31]	**10-40**	160[#32], 330[#31], 530[#33]	**300-500**	13[#32], 32.4[#18], 65[#33]	**30-70**		
**Vemurafenib**	4.03[#33], 23[#18, 20]	**1-20**	100[#20]	**100**	31[#34], 65[#18, 20]	**30-70**		
**Dabrafenib**	22[#20]	**20**	5.2[#35, 36]	**5**	0.8[#35, 36]	**1**		
**Sorafenib**	22[#37], 6.3[#20]	**1-20**	540[#20, 37]	**540**	260[#16]	**260**	520[#16, 37]	**520**

Table S1. The reported IC50 values of individual chemical drugs against the indicated kinases, related to Figure 1

	Name	5' to 3' sequence
Site-directed mutagenesis primer	canis sgZAK1#1_QC_primer_Fw	GGAAAGGACGAAACACCGATTATGAGATGTCGTCTCTGTTTTAGAGCTAGAAATAGC
	canis sgZAK1#1_QC_primer_Rv	GCTATTTCTAGCTCTAAAACAGAGACGACATCTCATAATCGGTGTTTCGTCCTTTCC
Genotyping primer	canis ZAK_geno-primer_exon1_Fw-1	GCCTTGCTTTCCTTTCTCCT
	canis ZAK_geno-primer_exon1_Rv-1	TGTGACCTCTGACTCGGTTG
	CK19-CreERT2 Fw	AATCGCCAGGAATTGACCAATGGGG
	CK19-CreERT2 Rv	CGCCCGTACCCCCAAAGGAAGACAT
	DNMT1-CAG-loxP-STOP-loxP-HRasV12-IRES-eGFP Fw	CACTGTGGAATCTCGGCAGG
	DNMT1-CAG-loxP-STOP-loxP-HRasV12-IRES-eGFP Rv	GCAATATGGTGGAAAATAAC
siRNA sequence	siRNA ZAK #1	GCUGCCUUCCUUUGAGAUUTT
	siRNA ZAK #2	GCUGGACUAAAGUGGAAUATT
qPCR primer	mouse ZAKα qPCR_primer_Fw1	acaccaagcatcaacccttc
	mouse ZAKα qPCR_primer_Rv1	gtttctggacagcccatactg
	mouse Actin qPCR_primer_Fw1	aaggccaaccgtgaaaagat
	mouse Actin qPCR_primer_Rv1	gtggtacgaccagaggcatac

Table S2. The sequences of primers and siRNAs used in this study, related to Figure 2, 3 and 4

TRANSPARENT METHODS

(EXPERIMENTAL MODEL AND SUBJECT DETAILS)

Experimental Animals

All animal experiments were conducted under the guidelines by the Animal Care Committee of Hokkaido University. The animal protocols were reviewed and approved by the Hokkaido University Animal Care Committee (approval number 12-0116). We used 6-10 weeks-old C57BL/6 mice for mating. Cytokeratin19 (CK19)-CreERT2 mice (Means et al., 2008) were crossed with DNMT1-CAG-loxP-STOP-loxP-HRasV12-IRES-eGFP mice (Kon et al., 2017) to create CK19-RasV12-GFP mice. Mice heterozygous for each transgene were used for experiments.

Cell Lines

MDCK and MDCK-pTR GFP-RasV12 cells were cultured as previously described (Hogan et al., 2009).

METHOD DETAILS

Cell competition-based high-throughput screening for chemical compounds using confocal microscopy

For the confocal microscopy-based primary screening, 5×10^3 MDCK-pTR GFP-RasV12 cells were mixed with 4.5×10^4 MDCK cells and seeded into a collagen-coated 96-well plate. The mixed cells were incubated at 37°C for 24 h until a monolayer was formed. Then, the culture medium was exchanged for new medium containing 10 μg ml^{-1} tetracycline and 10 μM each small chemical compound (Kinase inhibitor library I, SIGMA-ALDRICH), followed by further incubation for 16 h. Finally, cells were washed with PBS, fixed in 4% paraformaldehyde (PFA) /PBS, and stained with Hoechst 33342 and Alexa555-Phalloidin. The images of cells were captured by using a confocal imaging analyzer, IN CELL Analyzer 6000 (GE Healthcare). To evaluate the extrusion levels of GFP-RasV12 cells, the extruded GFP-positive cells from a cell monolayer were analyzed using a 65536-gradient step bright-field, and GFP-positive extruded cells were shown as white spots.

Antibodies and Materials

Chicken anti-GFP (ab13970) and rabbit anti-ZAK (ab65249) antibodies were purchased from Abcam. Rat anti-E-cadherin (131900) antibody was from Life Technologies. Anti-Paxillin (sc-5574) antibody was from Santa Cruz Biotechnology. Rabbit anti-phospho-MLC 2 (Thr18/Ser19; 3674) antibody was from Cell Signaling Technology. Mouse anti-Filamin (F6682) antibody was from Sigma-Aldrich. Mouse anti-Actin (MAB1501R) and mouse anti-HA (05-904) antibodies were from Merck Millipore. Alexa-Fluor-568- and -647-conjugated secondary antibodies were from ThermoFisher Scientific. Hoechst 33342 (Life Technologies) was used at a dilution of 1:5,000. For immunofluorescence, the primary antibodies described above were diluted in PBS containing 1% BSA at 1:100, except anti-Paxillin antibody at 1:50. All secondary antibodies were used at 1:200. Alexa-Fluor-568- and -647-conjugated Phalloidin (Life Technologies) were used at 1.0 U ml^{-1}. PLX4720, Vemurafenib, Dabrafenib, and Sorafenib were purchased by Chemscene LLC. DMSO (Sigma-Aldrich) was added as a control.

Cell Culture

MDCK and MDCK-pTR GFP-RasV12 cells were cultured as previously described (Hogan et al., 2009). To induce the expression of GFP-RasV12, the

tetracycline-inducible MDCK-pTR GFP-RasV12 cell lines were treated with 2 μg ml^{-1} tetracycline (Sigma-Aldrich). For the inhibitor treatment, the indicated inhibitors were added together with tetracycline, and cells were then cultured for 16 h or 24 h. For immunofluorescence, cells were seeded onto Type-I collagen-mounted coverslips as described below in the section of immunofluorescence.

CRISPR/Cas9-mediated generation of ZAK-knockout cells

Guide sequences of ZAK single-guide RNA (sgRNA) targeting canis ZAK were designed on exons 1 as described previously (Hsu et al., 2013). ZAK sgRNA sequences (ZAK sgRNA, 5'-ATTATGAGATGTCGTCTCT-3') were introduced into the pCDH-QC-sgRNA control (sgControl) vector (Maruyama et al., 2015) using primers listed in Table S2. First, MDCK cells were infected with lentivirus carrying pCW-Cas9 as described (Maruyama et al., 2015) and were cultured in the 500 ng ml^{-1} puromycin-containing medium. The tetracycline-inducible MDCK-Cas9 cells were transfected with the pCDH-QC-ZAK sgRNA (sgZAK) by nucleofection, followed by selection in medium containing 200 μg ml^{-1} hygromycin. The antibiotics-selected crude Cas9-sgControl or sgZAK MDCK cells were lysed with quick extraction buffer (Epicentre), and the lysates were subjected to PCR. The PCR amplicons were digested with Surveyor enzyme

(Integrated DNA Technologies). The crude cells were subjected to limiting dilution, and indels on the ZAK first exon in isolated monoclone were analyzed by direct sequencing using primers listed in Table S2. ZAK-KO cell lines which possess 2 base-homozygous depletion (KO1) or 17 base-homozygous insertion (KO2) were obtained. To generate ZAK-deleted cells carrying tetracycline-inducible GFP-RasV12, pPB-TRE3 GFP-RasV12 was introduced into the ZAK-deleted cells by nucleofection and antibiotic selection (Blasticidin, 5 μg ml^{-1}). In addition to the ZAK-KO MDCK-pTRE3G GFP-RasV12 cells, we generated ZAK-WT MDCK-pTRE3G GFP-RasV12 cells as a control cell line. To constitutively express HA-ZAK-WT or HA-ZAK-KN, MDCK-pTRE3G GFP-RasV12 ZAK-KO1 cells were transfected with PB-HA-ZAKα-WT or -KN and subjected to antibiotics selection (G418, 800 μg ml^{-1}).

Immunofluorescence

For immunohistochemical examinations of the small intestine and pancreas, the mice were perfused with 1% PFA (Sigma-Aldrich), and the isolated tissues were fixed with 4% PFA in PBS for 24 h and embedded in FSC 22 Clear Frozen Section Compound (Leica Biosystems). Then, 10-μm-thick frozen sections were cut on a cryostat. The sections were blocked with Block-Ace (DS Pharma

Biomedical) and 0.1% Triton X-100 in PBS. Primary or secondary antibodies were incubated for 2 h or 1 h respectively at ambient temperature. All primary antibodies were used at 1:1,000, and all secondary antibodies were at 1:500 except for ZAK antibody (1:100). For immunofluorescence of cultured cells, MDCK-pTR GFP-RasV12 cells were mixed with MDCK cells at a ratio of 1:50 and cultured on the collagen matrix as previously described (Hogan et al., 2009). The mixture of cells was incubated for 8-12 h until they formed a monolayer, followed by tetracycline treatment for 24 h. Cells were fixed with 4% PFA in PBS and permeabilized with 0.5% Triton X-100 in PBS, except for Filamin immunofluorescence for which cells were fixed in methanol at -20°C for 2.5 min as shown in Figures 3E and S3C, followed by blocking with 1% BSA in PBS. Alexa-Fluor-568- or -647-conjugated Phalloidin was incubated for 1 h at ambient temperature. Immunofluorescence images of mouse tissues and cultured cells were acquired using the Olympus FV1000 system and Olympus FV10-ASW software. Paxillin, ppMLC and Filamin-stained images were quantified with the ImageJ software.

***In vivo* mouse model and PLX treatment**

All animal experiments were conducted under the guidelines by the Animal Care Committee of Hokkaido University. The animal protocols were reviewed and approved by the Hokkaido University Animal Care Committee (approval number 12-0116). We used 6-10 week-old C57BL/6 mice for mating. Cytokeratin19 (CK19)-CreERT2 mice (Means et al., 2008) were crossed with DNMT1-CAG-loxP-STOP-loxP-HRasV12-IRES-eGFP mice (Kon et al., 2017) to create CK19-RasV12-GFP mice. Male and female mice heterozygous for each transgene were used for experiments. For PCR genotyping of mice, the sequence information of the used primers is shown in Table S2. Mice were age-matched and given a single intraperitoneal injection of 0.5 mg of tamoxifen (TAM) in corn oil (Sigma-Aldrich) per 20 g of body weight for the induction of RasV12 expression, and then sacrificed at Day 6 or Day 30 after Cre activation. To examine the effect of PLX4720, the mice were pretreated with an oral beverage of 300 mg l^{-1} PLX4720 at the post 1 day after TAM injection.

***In vivo* electroporation**

The iGT (intestine-specific gene transfer) using HVJ-E (haemagglutinating virus of Japan envelope) was modified and performed as follows (Imajo et al., 2015; Kon et al., 2017). Briefly, 3-5 cm regions of the small intestine drawn out from

the peritoneal cavity were tied with nylon string. 300 μl of the mucus removing solution (20 mM DTT and 0.05% Tween-20 in PBS) was injected into the intestinal lumen for 15 min, removed and incubated with the same solution for 10 min. After washing with PBS three times by pipetting, 300 μl of Opti-MEM (Thermo Fisher Scientific) containing Cy3-labelled siRNA was injected, and the tied part of intestine was sandwiched with electro-nodes (NEPAGENE; CUY650P5) for electroporation (condition: Voltage; 40 V, Pulse; 30 ms, Interval; 50 ms, 3 times, reduction; 10%). The sequences of ZAK-siRNA are listed in Table S2. 0.5 mg of TAM was injected into the peritoneal cavity when the wound was sutured. The mice were euthanized for analysis after 5 days of iGT.

Reverse Transcription and Quantitative PCR Analysis

Total RNA was extracted from isolated cell samples using Sepazol (Nacalai Tesque) and chloroform, precipitated with 2-propanol and washed with 75% (vol/vol) ethanol. RNA samples were incubated with DNase I (Invitrogen) to remove contaminating genomic DNA and then reverse-transcribed into cDNA (Superscript III reverse transcriptase, VIRO cDNA Synthesis Kit; Invitrogen). Quantitative PCR analysis was performed using LightCycler 480 II (Roche) with

FastStart Essential DNA Probes Master (Roche). Primer sequences are shown in the Table S2.

QUANTIFICATION AND STATISTICAL ANALYSIS

Two-tailed Student's *t*-tests or Chi-squared tests were used to determine *P*-values for statistical analyses. For quantification of the apical extrusion frequency, more than 100 transformed cells are subject to the apical extrusion analysis in each sample. For quantification of the immunofluorescence intensity, at least 30 transformed cells were analyzed using the ImageJ software for each experimental condition.
